# Antimycobacterial activity of fruit of *Zanthoxylum acanthopodium* DC against *Mycobacterium smegmatis*

**Published:** 2018

**Authors:** Heddy Julistiono, Fani Gustiani Lestari, Rifki Iryanto, Puspa Dewi Lotulung

**Affiliations:** 1 *Research Center for Biology - The Indonesian Institute of Sciences (LIPI). Jl. Raya Jakarta – Bogor Km 46, Cibinong 16911, Indonesia*; 2 *Research Center for Chemsitry - The Indonesian Institute of Sciences (LIPI). Tangerang Selatan, Banten 15314, Indonesia*

**Keywords:** Zanthoxylum acanthopodium, Mycobacterium smegmatis, Anti – diarrhea, Antimycobacteria, Geranyl acetate

## Abstract

**Objective:**

Fruits of lemon pepper (*Zanthoxylum acanthopodium* DC., Rutaceae) have been traditionally used as a spice and in folk medicine for treatment of diarrhea and stomachache. Stomachache could be associated with mycobacterial infection. The present study was designed to investigate the activity of *Z. acanthopodium* fruits against a non-infectious *Mycobacterium smegmatis* and to identify the important phytochemical constituent that is toxic towards mycobacteria.

**Materials and Methods:**

The broth microdilution method was used to determine the minimal inhibitory concentration (MIC) of ethyl acetate or hexane extract of green, young fruits of *Z. acanthopodium*. Effect of active extract (hexane) on cell membranee integrity was studied by measuring sodium and potassium leakage into extracelullar liquid using Atomic Absorbtion Spectrophotometer (AAS). Next, cell morphology was observed by using Scanning Microscope Electron (SEM). Column chromatography was used for fractionation and purification of hexane extract while the chemical structure of the active compound was determined using NMR technique. Rifampicin, an antimycobacterial compound, was used as positive control.

**Results:**

Hexane extract was active against *M. smegmatis* with an MIC of 64 µg/ml. Plant extract at the concentration of 128 µg/ml caused ions leakage. Concentration of sodium in extracellular liquid of cells treated with plant extract was significantly higher than that of untreated cells. SEM observation revealed cell wall deformation in cultures treated with the extract. NMR spectroscopy analysis of the most active fraction revealed that the compound that exerted toxicity on *M. smegmatis* was geranyl acetate.

**Conclusion:**

Geranyl acetate was an important constituent of *Z.* *acanthopodium* fruit that has antimycobacterial activity. Possibly, *Z. acanthopodium* fruit exert its toxic effects against *M. smegmatis* through damaging cell membrane.

## Introduction

Lemon pepper (*Zanthoxylum acanthopodium* DC., Rutaceae) or “andaliman” fruit has been traditionally used by Batak people in North Sumatera province, Indonesia (Yanti, et al., 2001 ▶). A preliminary study indicated that hexane extract showed potential inhibitory activity against pathogenic bacteria including *Salmonella typhimurium*, *Bacillus cereus*, and *Staphylococcus* *aureus*, as well as non-pathogenic bacteria (Parhusip et al., 2003 ▶). Gastrointestinal infections could be caused by viruses, bacteria, parasites, or other pathogens. In addition to bacterial infections, mycobacterial infections of the gastrointestinal tract are of crucial importance. Mycobacterial infections in the gastrointestinal tract of both immunocompromised and non-immunocompromised patients, were reported (Figueroa et al., 2016 ▶; Merali et al., 2015 ▶; Zhang et al., 2015 ▶)

Stomachache could be caused by non- tuberculosis or tuberculosis mycobacterial infections (Ding et al., 2016 ▶; Burke et al., 2014 ▶). The non-virulent and fast-growing *Mycobacterium smegmatis*, could be used as a surrogate host for pathogenic mycobacteria (Campen, 2015 ▶; Staudinger et al., 2014 ▶; Fan et al., 2015 ▶). Having similar characteristics with *Mycobacterium tuberculosis* make *M. smegmatis* a safe and convenient tool to study response of pathogenic bacteria to drugs. Also, close genetic relationship between *M. smegmatis* and two pathogenic mycobacteria *M. avium* and *M. tuberculosis* (Broxmeyer et al., 2002 ▶), supports the use of the nonvirulent mycobacterium to study physiological characteristic of the infectious ones. In this study we investigated the potential role of active substance of pepper lemon fruit against *M. smegmatis*. The data will reflect the role of this Indonesian traditional spice fruit in preventing stomachache caused by mycobacterial infection and its potential in combatting tuberculosis.

## Materials and Methods

**Plant Material**

Plant and unripe greenish fruits of lemon pepper were obtained from North Sumatera about 24 hours after fruits harvesting. Plant was identified and a voucher specimen (BO-0013731) was deposited at Herbarium Bogoriense (BO). Fruits were kept at 4 ºC overnight before extraction with n-hexane and ethyl acetate. 

**Chemicals**

Tween 80, dimethyl sulfoxide, cacodylate buffer, tannin acid, glutaraldehyde, potassium and sodium standard solutions, and HCl were purchased from Merck. DMSO was purchased from Amresco. Thiazolyl blue tetrazolium bromide (MTT), rifampicin, tetramethylsilane (TMS), and propan-2-ol were purchased from Sigma. Nutrient broth (NB) was purchased from HIMEDIA (India).

**Extraction, Fractionation and Identification of the Most Active Compound**

Green and fresh fruits (366 g) of *Z. canthopodium* were successively extracted using 1500 mL n-hexane and ethyl acetate. N- Hexane extract (3.11 g) was fractionated by column chromatography on silica gel using gradient. Column chromatography was carried out using Merck Silica gel 60 (70 - 230 mesh ASTM), and TLC (Thin Layer Chromatography). Active fraction was yellowish liquid that was identified using LC-MS and NMR (1H-NMR, 13C-NMR) spectrometer. NMR spectra were recorded using JEOL JNM ECA-500 spectrometer, operating at 500 MHz (1H-NMR) and 125.76, using TMS as an internal standard. Mass Spectrometry were obtained using a Mariner Biospectrometry using ESI (Electrospray Ionization) system and positive ion mode.

**Determination of hexane extract of lemon pepper fruit phytocompounds**

The extract of lemon pepper fruit were analyzed by using GC Agilent 7890 B tandem Mass Spectrometry, equipped with a 5977A detector, DB-Wax column (30 mm × 0.25 mm). The injector temperature was 250 ºC and the ion-source temperature was 150 ºC. Helium was used as the carrier at a constant flow rate of 1 ml/min. The oven temperature was programmed as follows: 40 ºC for 2 min, then heat up to 250 ºC (10 ºC/min) and maintained at 250 ºC for 5 minutes. Mass spectra were interpreted using the database of NIST and Willey. 

**Growth of the microorganism**

*M. smegmatis* NBRC 3082, obtained from InaCC (Indonesian Culture Collection), was maintained on slants of nutrient agar. These slants were used to inoculate 125-ml Erlenmeyer flasks containing 25 ml of nutrient broth (NB) (Hey-Ferguson and Elbein, 1970). After 72 hr of growth at room temperature, 1 ml of this culture was used to inoculate 300-ml Erlenmeyer flasks containing 100 ml of NB. 

**Minimum Inhibition Concentration (MIC)**

Extracts were dissolved in water containing 1 % (v/v) Tween 80. MIC was determined using the MTT assay (Moodley et al., 2014; El Baz and Shetaia, 2005). Aliquots of 50 μl of NB containing series of extracts or rifampicin (Sigma, R8626-1G) concentrations were added to each well of a 96-well plate (Iwaki). The concentrations ranged from 1 μg/ml to 256 μg/ml. Cell suspension (50 μl) was added to the appropriate wells (final OD at 600 nm value of 0.005) Plates were incubated at room temperature for 3 days. Thereafter, 10 μl of the MTT solution (5 mg/mL) was added to the wells followed by 2 hr incubation. To each tube, 11 μl of propan-2-ol containing 0.04 M HCl was added and incubated for another 2 hr. The absorbance of the cell suspension was measured at 595 nm. The MIC was defined as absorbance value of NB medium. 

**Cell membrane integrity observation**

Effects of the extract or rifampicin on the cytoplasmic membrane integrity of *M. smegmatis* were examined by sodium and potassium leakage assay according to the method of Suriyanarayanan et al. (2013) with some modifications. To small test tubes (13 × 100 mm) containing 1.5 ml of sterile water with 1 % (v/v) Tween 80; or Tween 80 and 128 µg/ml of extract; or Tween 80 and 2 MIC of rifampicin; *M. smegmatis *culture (OD 600=18.1) were added and incubated at room temperature while shaking at 100 rpm. A concentration of 128 µg/ml was applied to amplify the effect to become more detectable. Cells were collected after 24 and 72 hr. The samples were centrifuged at 15,000 g for 10 min. Supernatants of 1 ml were stored at – 20 °C for further analysis. Sodium and potassium ions concentrations were then measured using an Atomic Absorption Spectrophotometer (Shimadzu AA – 6800). Sodium nitrate and potassium nitrate were used as sodium and potassium standards, respectively.

**Scanning Electron Microscopy observation**

Samples were fixed using glutaraldehyde 2 % for 1 hr, followed by treatment with tannin acid 2 % for 2 hr, then washed with cacodylate buffer (pH 7.2). Finally, samples were fixed using 1 % OsO_4_ for 1 hr. Samples were dehydrated with graded ethanol series (30, 50, 70, 85, 95, and 100 %) for 10 min at each concentration. Cells were observed with SEM (Hitachi SU 3500) at electron energy of 10 kV.

**Statistical analysis**

Analysis of variance (ANOVA) was used, followed by Duncan’s test as the post hoc test to evaluate differences among means (SPSS ver 16). A p value less than 0.05 was considered statistically significant

## Results

**Activity of** **e** **xtract**

Hexane extract of *Z. acanthopodium* fruits had higher antimycobacterial activity compared to the ethyl acetate extract with an MIC of 64 µg/ml (Tabel 1). 

**Tabel 1:** Minimum Inhibitory Concentration (MIC) of lemon pepper fruit extracts.

**Sample**	**MIC (µg/ml)**
**Rifampicin**	8
**Hexane extract**	64
**Ethyl ** **a** **cetate extract**	> 256

**Phytochemical constituents of hexane extract of lemon pepper fruit**

Ten compounds were detected in the hexane extract of lemon pepper fruit (Tabel 2).

**Table 2:** Phytochemical constituents detected in the hexane extract of lemon pepper fruit

**No**	**Library**	**Quality**	**Relative Amount ** **(** **%)**
**1**	1-Decene	91	0.19
**2**	1-Tetradecene	99	0.75
**3**	Citronellal	97	0.12
**4**	Naphthalene	91	0.2
**5**	Geranyl acetate	91	3.56
**6**	Citronellol	98	0.30
**7**	Geraniol	94	1.29
**8**	2-Propenoic acid	96	0.37
**9**	Oleic Acid	99	5.44
**10**	Octadec-9-enoic acid	99	2.4

**Effect of the extract on ions leakage**

**Figure 1:**
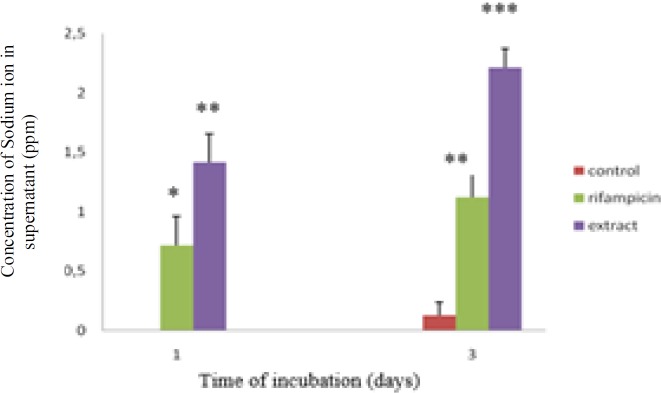
Effect of the extract on sodium leakage. Values are expressed as mean±SE (n=3). * p<0.05 indicates a significant difference as compared to control (One-way ANOVA).

**Figure 2:**
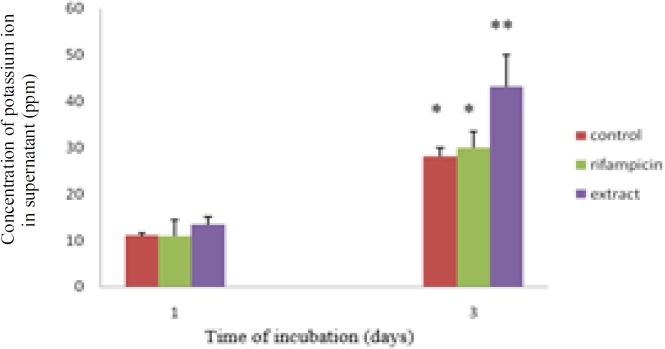
Effect of the extract on potassium leakage. Values are expressed as mean±SE (n=3). *p<0.05 indicates a significant difference as compared to control (One-way ANOVA followed by Duncan’s test).

Figure 1 shows sodium leakage in cells treated with 128 µg/ml of the extract or 16 µg/ml of rifampicin after 1 and 3 days of incubation. Sodium leakage was more marked in cells treated with the extract. Potassium leakage was also observed in cells treated only with the extract after 3 days of incubation (Figure 2). 

**Effects of the extract fractions against** ***M. smegmatis***

**Figure 3:**
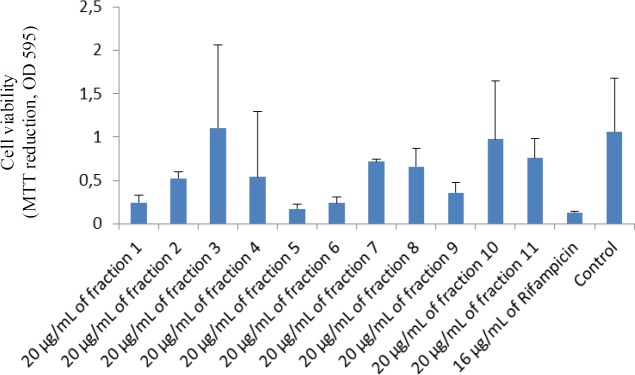
Antimycobacterial activity of different fractions of the extract against *M. smegmatis*. Bars represent mean±standard deviation of three independent experiments.

As shown in Figure 3, fraction 5 had the most pronounced activity against *M. smegmatis*, compared to other fractions. The main compound was then found in fraction 5.

**Morphology of cells treated with the extract**

**Figure 4:**
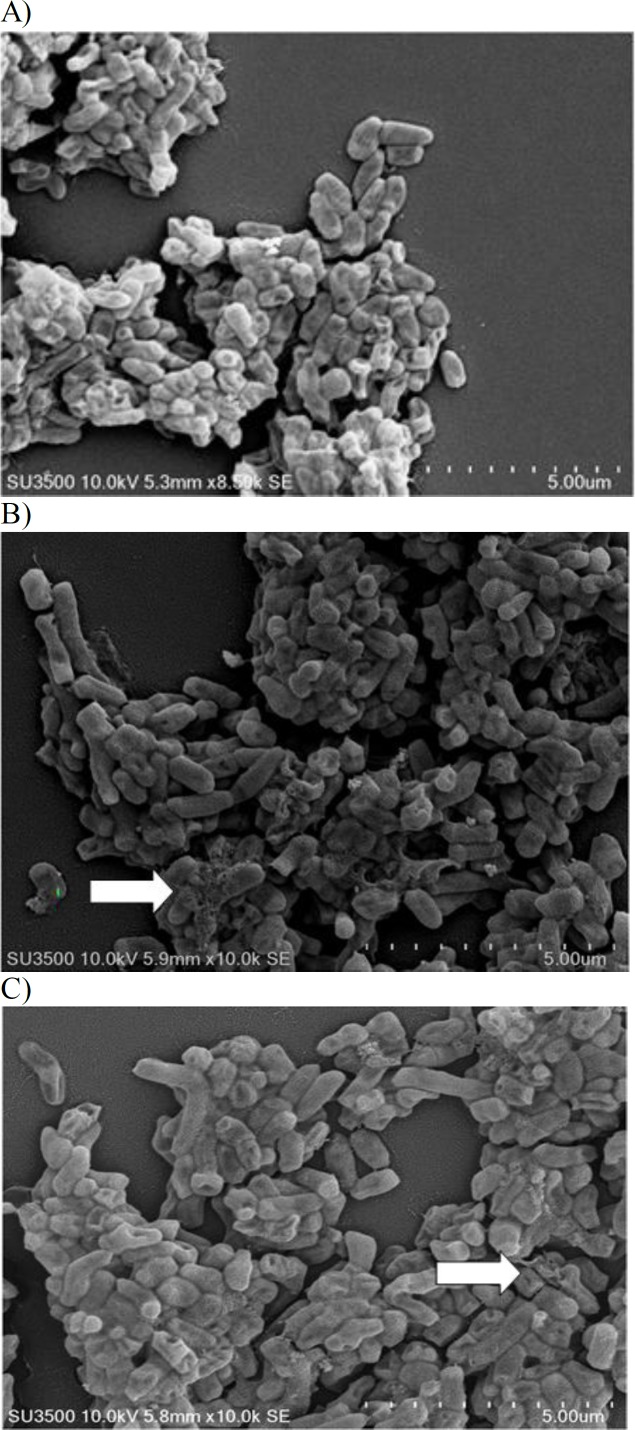
A) Morphology of untreated cells. B) Morphology of cells treated with rifampicin. The arrow indicates degraded cells. C) Morphology of cells treated with the extract of *Z*. *acanthopodium* fruit. The arrow indicates degraded cells.

SEM was used to examine morpholgical change in cells following treatment by plant extract or rifampicin. In the untreated sample, the cells looked still intact and no lysed cell with cell debris was observed, while in extract- or drug- treated samples, we observed some cells with craters in their cell wall. Some cells that were treated with rifampicin (Figure 4B) or the extract (Figure 4C), were damaged reflecting cell membrane disintegrity whereas untreated cells were still intact (Figure 4A). Figure 4 B shows that some cells treated with the extract were more severely damaged compared to those treated with rifampicin.

**Identification** **of the main** **Compound**

**Figure 5:**
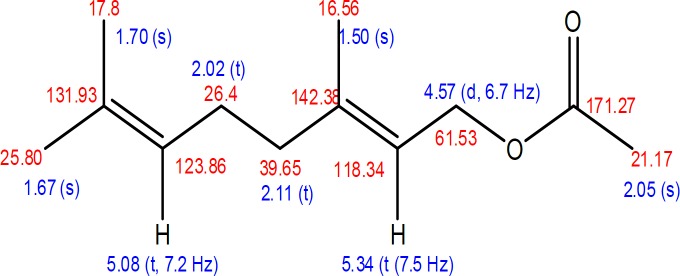
Structure of geranyl acetate and its chemical shift data.

The ^1^H-NMR (CDCl_3_, 500 MHz) spectrum data indicated the presence of 3 singlet methyls at δ_H_ 1.50, 1.67 and 1.170, acetyl group at 2.05 (s) and two olefinic protons at δ_H_ 5.08 (t, J=7.2 Hz), 5.34 (t, J=7.5 Hz). The other signals are methylene groups appeared at δ_H_ 2.04 (t, J=7.2 Hz), 2.11 (t, J=7.2 Hz) and 4.57 (d, J=6.7 Hz). That was also supported by the ^13^C spectrum (125 MHz) data as 3 methyls found at δ_C_ 16.95 (q), 17.18 (q), 21.17 (q); one acetyl at δ_C _25.80, three proton methyenes at 26.4 (t), 39.65 (t) and 61.53 (t); double bonds at δ_C_ 118.34 (d), 123.86 (d), 131.93 (s) and 142.36 (s), in the spectrum also showed the presence of carbonyl (ester) at δ_C_ 171.27 (s). The NMR data was summarized in the structure of geranyl acetate (Figure 5). Also, geranyl acetate identity was confirmed by its molecular weight (i.e. 196.2877 g/mol) with the presence molecular ion at 219.2877 [M+Na] ^+^ in the mass spectrum. It was also supported by the NMR data of geranyl acetate reported in the literature (Chakraborti and Gulhane, 2003 ▶).

## Discussion

Plants are prosmisng sources of antimycobacterial molecules for combating drug resistance (Singh et al., 2015 ▶). In certain cases, mycobacterial infection can occur in the gastrointestinal tract (Figueroa et al., 2016 ▶; Merali et al., 2015 ▶; Zhang et al., 2015 ▶). It is intriguing to understand the role of lemon pepper fruit in protecting gastrointestinal tract against mycobacterial infection. By using a non-virulent mycobacteria, we demonstrated that a traditionl spice, lemon pepper, can combat against mycobacterial infection. Geranyl acetate present in this fruit is responsible for the antimycobacterial activity. Another terpene compound, geranylgeranyl acetate was also found to be a selective inhibitor of *M. tuberculosis* (Vik et al., 2007 ▶). Several researchers reported the activity of monoterpenes against gram positive and negative bacteria (Nazzaro et al., 2013 ▶). Lipophilicity and water solubility of monoterpenes lead to perturbation of the lipid fraction of microorganism plasma membrane causing alterations in membrane permeability and leakage of intracellular materials (Trombetta et al., 2005 ▶). We observed cell membrane dammage (Figure. 4C) and ions leakage (Figures 1 and 2) in *M. smegmatis* treated with the extract. The data tends to suggest that antimycobacterial activity of this extract may be due to its effects on the cell membrane. It is intriguing to study the interactions between geranyl acetate and mycobacterial cell membrane in order to better understand how this active compound affect the membrane cell. However, other essential compounds of the fruit (presented in Table 2) could also be responsible for the activity agaisnt *Mycobacterium* such as the oleic acid that was reported to have antimycobacterial activity against *M. smegmatis* and *M. tuberculosis *(Saravanakumar et al., 2008 ▶).

Free radicals may also be another action of a drug to induce cell membrane alterations. Gupta et al (2012) ▶ reported that *M. tuberculosis* cell membranee was damaged due to lipids peroxidation caused by oxidative stress. Rifampicin induced hydroxyl radical formation in *M. tuberculosis *(Piccaro et al., 2014 ▶). Possibly, in this study, rifampicin may induce reactive oxygen species formation that attacks cell membrane.

Results from this study show that fruits of *Z. acanthopodium*, a traditional medicinal plant used against stomachache or diarrhea, has potent activity against mycobacteria. Geranyl acetate is an important compound in the fruit that kills mycobacteria. 
